# Machine learning based combination of multi-omics data for subgroup identification in non-small cell lung cancer

**DOI:** 10.1038/s41598-023-31426-w

**Published:** 2023-03-21

**Authors:** Seema Khadirnaikar, Sudhanshu Shukla, S. R. M. Prasanna

**Affiliations:** 1 Department of Electrical Engineering, Indian Institute of Technology Dharwad, Dharwad, India; 2 Department of Biosciences and Bioengineering, Indian Institute of Technology Dharwad, Dharwad, India

**Keywords:** Cancer, Computational biology and bioinformatics, Oncology

## Abstract

Non-small Cell Lung Cancer (NSCLC) is a heterogeneous disease with a poor prognosis. Identifying novel subtypes in cancer can help classify patients with similar molecular and clinical phenotypes. This work proposes an end-to-end pipeline for subgroup identification in NSCLC. Here, we used a machine learning (ML) based approach to compress the multi-omics NSCLC data to a lower dimensional space. This data is subjected to consensus K-means clustering to identify the five novel clusters (C1–C5). Survival analysis of the resulting clusters revealed a significant difference in the overall survival of clusters (*p*-value: 0.019). Each cluster was then molecularly characterized to identify specific molecular characteristics. We found that cluster C3 showed minimal genetic aberration with a high prognosis. Next, classification models were developed using data from each omic level to predict the subgroup of unseen patients. Decision‑level fused classification models were then built using these classifiers, which were used to classify unseen patients into five novel clusters. We also showed that the multi-omics-based classification model outperformed single-omic-based models, and the combination of classifiers proved to be a more accurate prediction model than the individual classifiers. In summary, we have used ML models to develop a classification method and identified five novel NSCLC clusters with different genetic and clinical characteristics.

## Introduction

Non-small cell lung cancer (NSCLC) with three subtypes, namely, squamous-cell carcinoma (LUSC), adenocarcinoma (LUAD), and large-cell carcinoma contributes to the majority of the lung cancer-related deaths every year^1^. It is projected that in the US alone, for the year 2022, there will be 1,918,030 new cancer cases^1^. Lung cancer alone will contribute to 236,740 new cases (both sexes combined) and will be a leading cause of cancer related deaths^1^. The first line of treatment for lung cancer is determined based on the histopathological stage and includes chemotherapy, surgery, radiation, targeted therapy, and their combinations^2^. Even with the advancements in therapies, the 5-year survival rate for lung cancer remains minimal^1^. The poor survival rate can be attributed to the ineffectiveness of the first line of therapy due to the lack of understanding of underlying tumor heterogeneity at the molecular level^2–5^. The heterogeneity of the tumor is largely determined by the genetic and epigenetic makeup of the tumors^6,7^. Therefore, precise identification of the molecular subtypes (subgroups) using molecular data is essential in order to effectively use the existing treatment strategies and improve the patient care^3^.

With the rapid development of high-throughput sequencing (HTS) technologies, massive amounts of molecular data are being generated at various levels of evidence (single-omic level)^8,9^. Projects like The Cancer Genome Atlas (TCGA) have successfully used the HTS technologies to generate genomic, epigenomic, transcriptomic, and proteomic data to characterize cancer and normal samples across 33 cancer types^10^. Several studies have attempted subgroup identification using the TCGA data. The initial studies used statistical methods to develop models for subgroup identification and prognosis^11–13^. As these studies are based on single-omic, they do not take into account the inter-dependencies between different omics.

It is necessary to consider information from multiple levels of evidence while subgrouping to model complex biological phenomena^14,15^. Besides providing additional information, adding multiple levels of evidence will increase the dimension of the data. In the case of machine learning (ML) models, the large dimension of the data may lead to overfitting due to the relatively small number of samples^16^. To overcome this, first, the large-dimension data needs to be converted into a lower dimension. This can be done using linear projection approaches like principal component analysis (PCA). However, disease phenotype is the resultant of a combination of genetic and epigenetic factors which may not be linear^17,18^. Therefore, ML techniques can be used to integrate different levels of evidence and project it to a lower dimension in a non-linear manner using models like autoencoders (AE)^19^.

Several attempts have been made to use multi-omics data for various applications, including patient stratification^16,20,21^. Chaudray et al. made one of the early attempts in the direction of early data integration using ML in cancer to predict the survival in hepatocellular carcinoma (HCC) samples using mRNA, miRNA, and methylation data^20^. The authors identified prognostic subgroups with a significant difference in survival by explicitly applying Cox-regression as the loss function to retain the features contributing to survival. Baek et al. carried out their work in the same direction on pancreatic cancer (PAAD) using mRNA, miRNA, and methylation data to cluster the patients^16^. Here, mutation data along with multi-omics data and clinical data is used to build a classification model to predict the five-year recurrence and survival. Recently, Zhan et al. combined the information from histopathology images (H and E) and transcriptomic data to predict the survival in HCC patients^22^. They proved that imaging based predictions are more accurate than Cox-PH based predictions alone.

All these works demonstrated that multi-omics data conveys more information than single-omic. We hypothesize that addition and non-linear processing of distinct levels of information will further improve the discriminative ability. In this work, in addition to mRNA, miRNA, and DNA methylation data, protein expression data is also integrated. Proteins have a crucial role to play in cellular signaling and phenotype determination^23,24^. Expression patterns of proteins carry vital diagnostic and prognostic information^25^.

Besides survival prediction as done in^16,20,22^, multi-omics data integration strategy can also be used for subgroup identification. Several studies have discussed the significance of subgroup identification from the point of view of precision therapy^3^. One of the important directions in the application of ML to multi-omics data is to use it for the identification of the subgroup to which the samples belong. This will help the clinicians decide on the treatment regimen. Our aim in this work is to identify the novel molecular subgroups in NSCLC to convey additional information, besides the existing histopathological grades. This additional information about subgroups will help in the effective utilization of the existing treatment strategies. Also, we aim to build classification models to predict the class labels for new samples. The final classification label will be obtained in two steps. In the first step, the most widely used classification models, support vector machine (SVM), Random forest (RF), and feed-forward neural network (FFNN) ($L_0$), will be used to obtain the prediction probabilities. As each of these classification models are based on different principles, the prediction probabilities will be concatenated and used as input to train the decision-level fused classifiers ($L_1$). The decision-level fused classifiers include linear and non-linear (logistic regression and FFNN) classification models^26–28^. As different levels of evidence convey complementary information, classification models will be built based on the feature-level fusion technique. In these models, the features originating from different omic levels will be fused to obtain a single representation which in turn will be used to train the classification models^17,29^. The features from different levels of evidence will be concatenated to obtain the fused feature representation and train the classification models.

**Figure 1:**
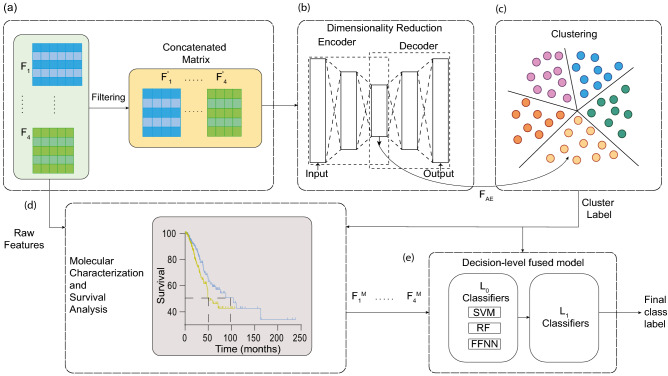
Overall pipeline followed in this work. (**a**) Each level of evidence (single-omic) was preprocessed and multi-omics representation was obtained by stacking the features for feature-vectors (samples) common across them. (**b**) The latent representation of multi-omics data (F$_{AE}$) was obtained using an autoencoder (AE). (**c**) Consensus *K*-means clustering was applied on the reduced dimension representation to obtain the cluster labels. (**d**) Molecular characterization of samples in clusters obtained was carried out to understand the subgroups. (**e**) Decision-level fused classifiers obtained by the combination of classification models including, support vector machines (SVM), random forest (RF), and feed-forward neural network (FFNN) was proposed for subgroup identification.

## Results

The overview of various steps involved in this work are outlined in Fig. 1. An outline of the steps followed for preprocessing the mRNA (F1), miRNA (F2), methylation (F3), and protein expression (F4) data is shown in Supplementary Figure S1. The details of the data used for subsequent analysis is summarized in Supplementary Table S1.

**Figure 2:**
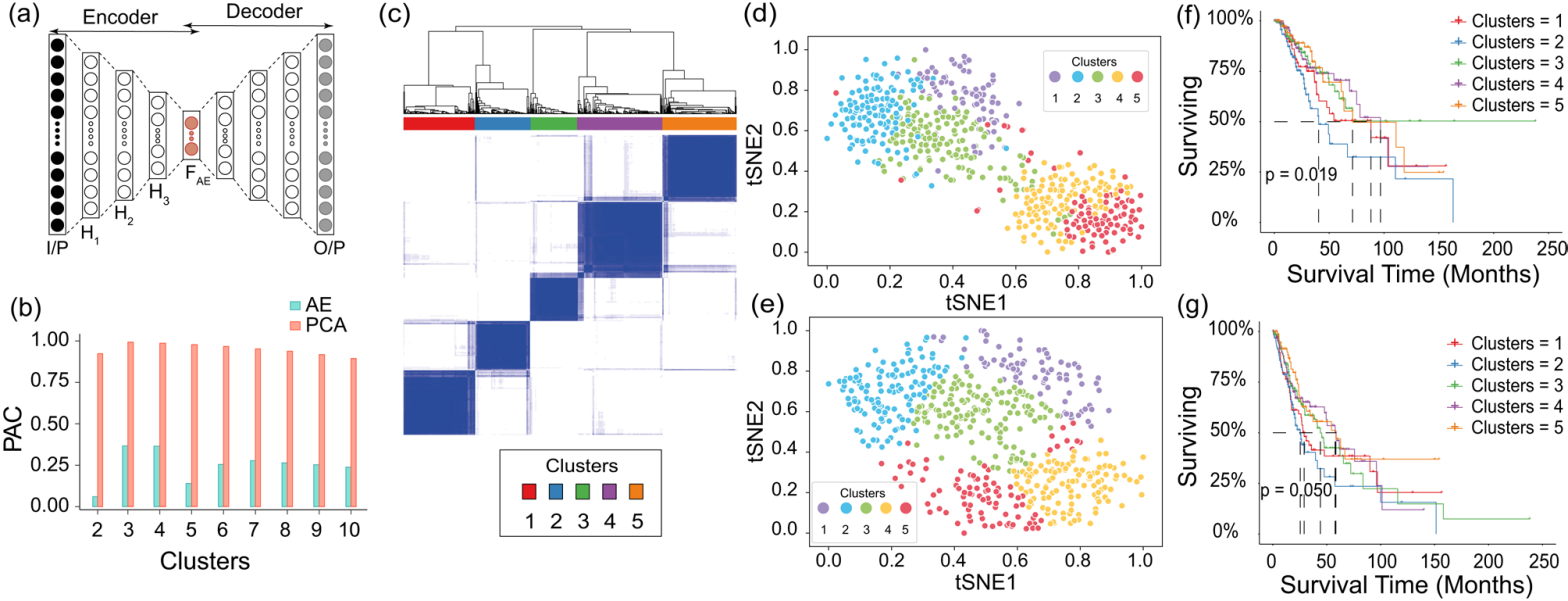
(**a**) Architecture of the autoencoder (AE) used in this study. Here, H$_1$, H$_2$, and H$_3$ are the first, second, and third hidden layers with 2000, 1000, and 500 nodes, respectively. F$_{AE}$ is the encoded representation from the bottleneck layer with 100 nodes. (**b**) Proportion of ambiguously clustered pairs (PAC) values obtained from the CDF curve for consensus clustering of reduced dimension data obtained from AE and PCA. (**c**) Consensus clustering heatmap for *K* = 5. (**d**) and (**e**) t-SNE plots for samples in original dimension, and reduced dimension obtained using AE. Samples are colored based on the labels obtained by consensus *K*-means clustering. (**f**) and (**g**) Kaplan-Meier plots for overall (OS) and disease-free survival (DFS) in the clusters obtained by consensus *K*-means clustering.

### Dimensionality reduction and clustering

In this work, an under-complete autoencoder (AE) with three hidden layers, each with 2000, 1000, and 500 nodes, and bottleneck layer with 100 nodes was used (Fig. 2 a, and Supplementary Figure S2). This architecture was chosen as it had the least difference between training and validation losses (Supplementary Table S2). The reduced dimension multi-omics representation from AE was clustered, and the proportion of ambiguously clustered pairs (PAC) values were obtained using Eq. (1) with $u_{1}=0.1$ and $u_{2}=0.9$ (Supplementary Figure S3 a and Fig. 2b). Although the least PAC value was obtained for $K=2$ (PAC = 0.06), the clusters here represented the two known histological NSCLC subtypes, LUAD and LUSC (Supplementary Figure S3b and c). Hence, the subsequent smallest PAC value was examined. As the cluster with $K=5$ had the next smallest PAC value (PAC = 0.14), the cluster labels obtained for this case were considered for subsequent analysis. Besides having a small PAC value, the consensus heatmap for $K=5$ was also consistent (Fig. 2c).

To visualize the distribution of samples in these five clusters, both before and after dimensionality reduction by AE, t-SNE plots were generated. It was evident from the t-SNE plots that there was a significant overlap between the samples in the original feature space (Fig. 2d). Also, the samples can be distinguished with minimal overlap when the dimension of the data was reduced using AE (Fig. 2e). We also used UMAP to visualize the sample distribution and found it to be similar to t-SNE (Supplementary Figure S4)^30^.

The PAC value obtained by clustering the multi-omics data without dimensionality reduction by AE (PAC = 0.31) was higher as compared to the case of dimensionality reduction by AE (PAC = 0.14) (Table 1). This observation indicated that the AE model was able to combine and capture the variation of information in the muti-omics data, and dimensionality reduction is an essential step in obtaining consistent clusters.

Additionally, we compared our AE based technique with the widely used unsupervised linear dimensionality reduction technique, principal component analysis (PCA). The top 100 principal components (PCs) were obtained by applying PCA on the multi-omics data matrix (standardized by mean and standard deviation). These PCs were then clustered using consensus *K*-means clustering. The number of clusters was varied from 2 to 10. The PAC values thus obtained were consistently high (closer to 1). This indicated that none of the clusters obtained were consistent (Fig. 2b, PAC = 0.98 for $K= 5$). This result validates the hypothesis that non-linear dimensionality reduction is required for biological data, which has also been shown in earlier studies^31^.

We also carried out the clustering of the subset of selected features from individual levels of evidence (single-omic) and their combinations. Clustering was carried out on these chosen features with and without dimensionality reduction by AE and PCA (Table 1). The PAC values obtained for these cases were higher than the multi-omics case (with all the four factors combined). This result signifies that the multi-omics clusters were more consistent than single-omic. Also, multi-omics with protein expression (F4) had smaller PAC value (PAC = 0.14) when compared to the combination of mRNA (F1), miRNA (F2), and methylation (F3) only (PAC = 0.28) (Table 1). This observation supported the hypothesis that protein expression indeed has a significant role to play in addition to other omics. Hence, strengthening the assumption that the combination of different omics conveys more information than the individual levels of evidence.

**Table 1:** Summarizing the PAC values obtained for *K* = 5 for each level of evidence for the subset of selected features, when clustered without dimensionality reduction, and with dimensionality reduction using PCA and AE (F1: mRNA (PcGs) expression, F2: miRNA expression, F3: DNA methylation, F4: protein expression).

Omic	Dimension	Without dimensionality reduction	With dimensionality reduction	
			PCA	AE
F1	2000	0.36	0.98	0.37
F2	407	0.46	0.97	0.49
F3	2000	0.25	0.94	0.25
F4	216	0.43	0.97	0.44
F1 + F2	2407	0.35	0.95	0.42
F2 + F3	2407	0.26	0.94	0.24
F1 + F3	4000	0.35	0.98	0.28
F1 + F2 + F3	4407	0.36	0.96	0.28
F1 + F2 + F3 + F4	4623	0.31	0.98	0.14

Further, we compared the proposed technique with *iClusterPlus*^32^, an existing and widely used statistical multi-omics data integration technique^33–35^. *iClusterPlus* was applied to multi-omics data, and the parameters were tuned using *tune.iClusterPlus* as recommended by the authors. The clusters obtained using our technique, and *iClusterPlus* were compared using two cluster evaluation methods, Silhouette coefficient, and Calinski-Harabasz index. The closer the value of the Silhouette coefficient to one and the higher the Calinski-Harabasz index, the better is the clustering. Both these scores indicated that the clusters obtained using the proposed algorithm were better separated than *iClusterPlus* (Supplementary Table S3). These evaluation measures were also computed to compare the consensus *K*-means clustering with hierarchical clustering (HC), Gaussian mixture models (GMM), and regular *K*-means clustering algorithm. The clustering scores obtained for consensus *K*-means and regular *K*-means were comparable in this case (Supplementary Table S4). But literature shows that consensus clustering outperforms regular clustering techniques^33,36^.

In addition, we performed the ablation study by varying the number of features from F1 and F3, and evaluated the performance of the AE model. The number of input features from F1 and F3 levels were varied (from 1000 to 4000), and the complete pipeline was repeated for different architectures of AE’s. The performance was compared using the PAC values for $K=5$ in each of the cases (Supplementary Table S5). It was observed that the PAC value was smallest when the top 2000 most varying features were considered from F1 and F3.

### Clinical and biological characterization of clusters

To understand the clinical significance of the different clusters obtained, we compared the survival times among the five clusters (Fig. 1d). The comparison of survival time using the log-rank test showed a significant difference in the survival of the patients (OS p: 0.019 and DFS p: 0.050). This suggests that there was at least one group whose survival was significantly different from the rest. Further, we used Kaplan-Meier (KM) plots to visualize the difference in the survival curves. We observed that the patients in Cluster 2 (C2 median survival 40.37 months) had significantly lower overall survival (OS). In comparison, patients in Cluster 3 (C3 median survival not reached i.e., more than half of the samples did not experience the event (death)) had the best OS rate. Patients in Cluster 1 (C1), Cluster 4 (C4), and Cluster 5 (C5) showed intermediate OS (Fig. 2f). This observation was also true for DFS (Fig. 2g). The survival analysis of the clusters obtained via PCA did not yield a significant difference in survival time (OS p: 0.169 and DFS p: 0.446). This indicates that the groups obtained were not clearly separable. This is in phase with the conclusion drawn based on the PAC value as well, that the clusters obtained via PCA were inconsistent. This also validates the consistency of our method over PCA.

**Figure 3:**
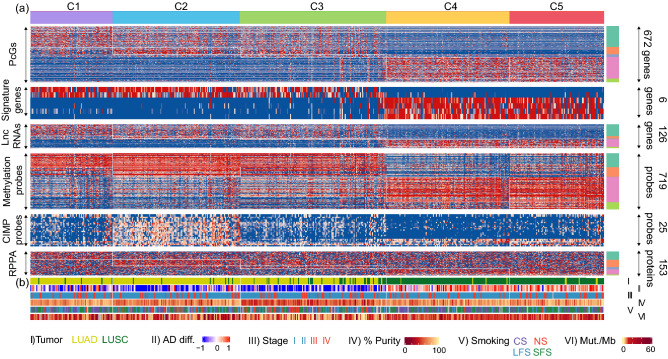
Characterization of different molecular levels of evidence. (**a**) Heatmap indicating the expression of protein coding genes (PcGs), LUAD-LUSC signature genes (NKX2-1, KRT7, KRT5, KRT6A, SOX2, TP63), long non-coding RNAs (lnc RNAs), CpG probes, CIMP probes, and protein expression in the subgroups obtained by multi-omics clustering. (**b**) Heatmap showing TCGA subtype, AD differentiation, pathological stage, tumor purity, smoking status (NS, lifelong never-smokers; LFS, longer-term former smokers greater than 15 years; SFS, shorter-term former smokers; CS, current smokers), and mutation rate in the multi-omics subgroups.

The differences in survival might be the resultant of underlying genetic and epigenetic variation among the clusters. To understand the molecular differences among the clusters, and to identify the molecular features specific to each subgroup, we compared the mRNA, miRNA, DNA methylation, and protein expression among the newly identified clusters (Fig. 3 and Supplementary Figure S5). We identified 672 PcGs that were differentially expressed across the five clusters (Supplementary Table S6 and Fig. 3a). Network analysis using the differentially expressed genes identified important biological pathways that were regulated, specifically in each cluster type (Supplementary Table S7). Further, we also identified 127 long non-coding RNAs (LncRNAs), nine miRNAs, and 719 CpG probes as differentially expressed (Supplementary Table S6 and Fig. 3a). The clinical characteristics including lung cancer subtype (LUAD and LUSC), the AD differentiation^37^, patient stage, tumor purity^38^, smoking status (NS: never smokers; LFS: long-term smokers greater than 15 years; SFS: shorter-term smokers; CS: current smokers) and mutation rate were obtained from Chen et al. study^33^ (Fig. 3b). It showed that patients in cluster 3 had a lower mutation rate and lower purity, i.e., a lower proportion of tumor cells in the tumor microenvironment.

**Figure 4:**
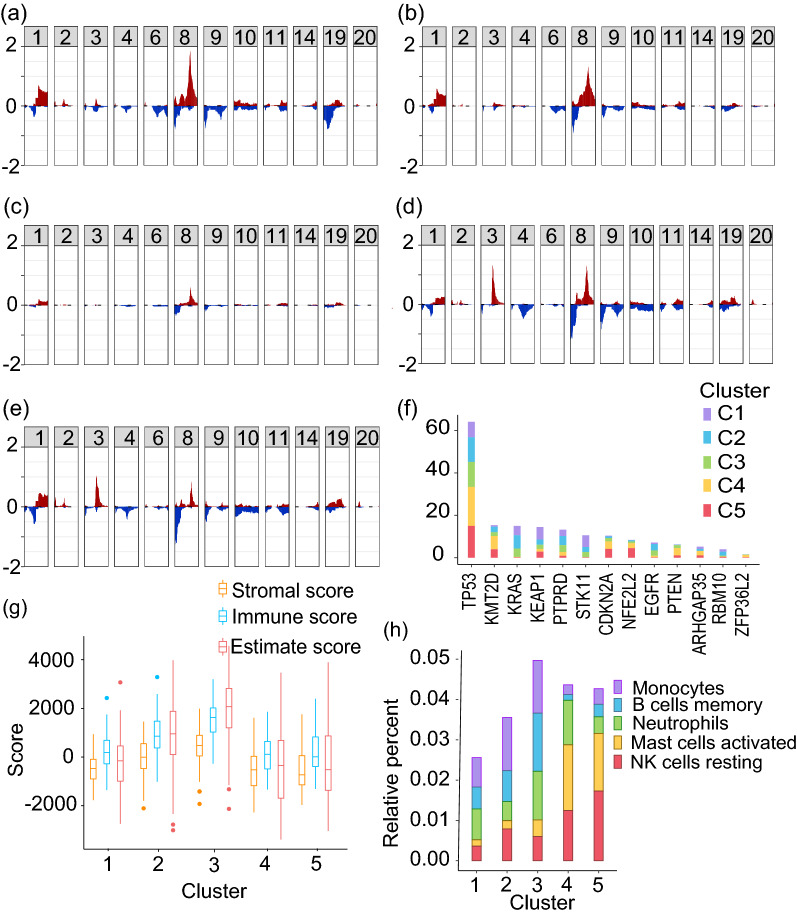
Molecular characters of samples with class labels obtained using consensus *K*-means clustering. (**a**)–(**e**) Frequency plots for copy number variation corresponding to clusters 1–5 (y-axis: proportion of copy number gain/loss, x-axis: Chromosome number) and (**f**) Mutation of driver genes in the subgroups. (**g**) Box plot showing the distribution of stromal, immune, and ESTIMATE scores in each subgroup. (**h**) Bar plot showing the distribution of significantly enriched immune cell types in the subgroups.

Furthermore, to understand the genetic differences and to identify the significantly different driver genes, we compared the CNV and mutation among the clusters (Fig. 4a–f). The steps followed for these analysis are outlined in Supplementary Figure S5^33,39^. C1 had significantly higher focal amplification of Chr 8 (8q24.21, q = 0.004) and Chr 1 (1q21.3, q = 0.001) (Fig. 4a). C2 also had amplification of Chr 8(8q24.21), and C4 of Chr 3 (3q26.33) and Chr 8 (8p11.23, q = 0.001) (Fig. 4b and d). C5 has significantly higher focal deletion of Chr 8 (8p23.2, q = 0.002) (Fig. 4e). As expected, TP53 had a higher mutation rate in all clusters compared to other genes. Cluster 1 (C1) had higher mutation of KEAP1 (q = 0.020), KRAS (q = 0.020), and STK11 (q = 0.020). EGFR was most mutated in cluster 2 (C2) (q = 0.020), PTEN in cluster 4 (C4) (q = 0.020), and CDKN2A in cluster 5 (C5) (q = 0.020) (Fig. 4f). Interestingly, cluster 3 (C3) had a lower mutation rate and copy number alteration as compared to other subgroups (Fig. 4c, Supplementary Table S8).

Tumor growth, invasion, and metastasis is largely determined by the tumor microenvironment (TME)^40,41^. The infiltration of different immune cells also defines the clinical and biological nature of the cancers. Hence, we performed ESTIMATE analysis in the newly identified subgroups of the NSCLC patients^42^. The ESTIMATE analysis showed the highest infiltration of immune cells in C3 (Fig. 4g). To understand the infiltration of individual immune cell types, CIBERSORT analysis was carried out using the LM22 signature gene set^43^. The CIBERSORT results further confirmed the ESTIMATE analysis results with the highest enrichment of monocytes, B cells, and neutrophils in C3 (Fig. 4h). Further, to understand the pathways enriched in C3, Gene Set Enrichment Analysis (GSEA) was carried out using the signature gene sets obtained from MSigDB^44,45^. The GSEA analysis of C3 vs. rest, carried out using the hallmark gene sets, showed significant enrichment of immune-related pathways in C3 (Supplementary Table S9 and S10).

### Subgroup identification by classifier combination

**Figure 5:**
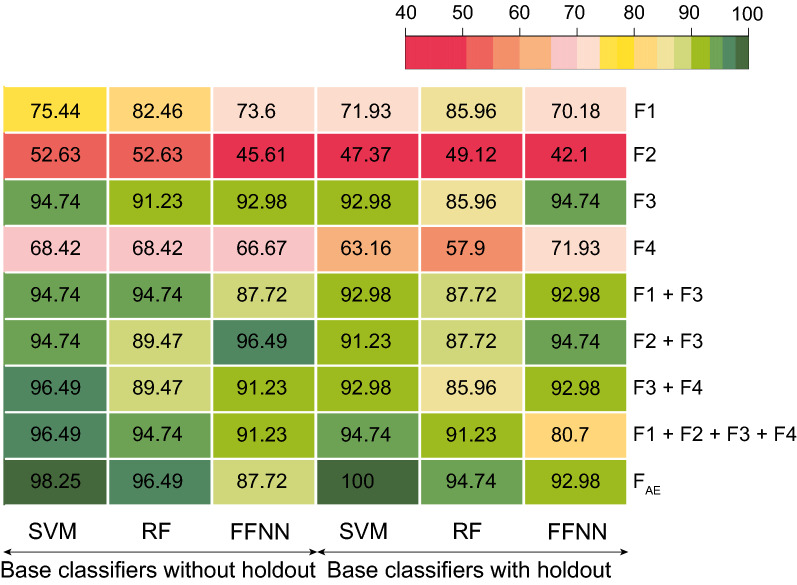
Classification accuracy of different base classifiers tested on different omic-levels and their combinations (F1: mRNA (PcGs) expression, F2: miRNA expression, F3: DNA methylation, F4: protein expression, F$_{AE}$: features from bottleneck layer of autoencoder, SVM: support vector machine, RF: random forest, FFNN: feed-forward neural network).

To help in the identification of class labels for a new sample, decision-level fused classification models were built. Each level of evidence is known to convey different information controlling different aspects of phenotype^17,29^. Hence, the classification models were trained using each molecular level of evidence. Based on the classification accuracy obtained on the test data set, it was observed that F3 (DNA methylation) had the highest classification accuracy for both base classifiers ($L_0$) and decision-level fused models ($L_1$) (Table 2, Fig. 5, and Supplementary Figure S6).

As each level of evidence conveys complementary information, classification models were also obtained for the feature representation obtained by fusing features from different levels of evidence. F3 was combined with other levels as it had the highest classification accuracy at the single-omic level. It can be observed from Table 2 that the decision-level fused classifier trained with feature-level fused molecular features from F3 and F4 had the highest classification accuracy among all the decision-level fused models. The presence of a small number of samples to train the learners might be one of the reasons for the poor performance of the non-linear decision-level fused model over the linear decision-level fused model. The classification models were also built for the combination of features from all four factors. But there was no improvement in accuracy as compared to the combination of F3 and F4. We also trained the classification models with the reduced dimension features obtained from the AE. We observed that the classification accuracy was highest for these features (Table 2). Hence, we concluded that the AE was able to capture the variation present in the multi-omics data effectively.

**Table 2:** Summarizing the test accuracy from different classifier combination techniques for different levels of evidence (F1: mRNA (PcGs) expression, F2: miRNA expression, F3: DNA methylation, F4: protein expression, F$_{AE}$: features from bottleneck layer of autoencoder, LR: logistic regression, FFNN: feed-forward neural network).

Omic	Dimension	Decision-level fused model w/o holdout			Decision-level fused model with holdout	
		Linear	LR	FFNN	LR	FFNN
F1	672	85.97	73.69	77.19	70.18	70.18
F2	9	89.00	50.88	50.88	49.12	52.63
F3	719	92.98	92.98	94.74	96.49	96.49
F4	153	68.42	70.18	57.90	70.18	61.40
F1 + F3	1391	94.74	92.98	94.74	92.98	92.98
F2 + F3	728	94.74	96.49	94.74	94.74	94.74
F4 + F3	872	98.25	96.49	98.25	96.49	96.49
F1 + F2 + F3 + F4	1553	98.25	96.49	98.25	91.23	91.23
F$_{AE}$	100	100.00	98.25	100.00	100.00	100.00

**Figure 6:**
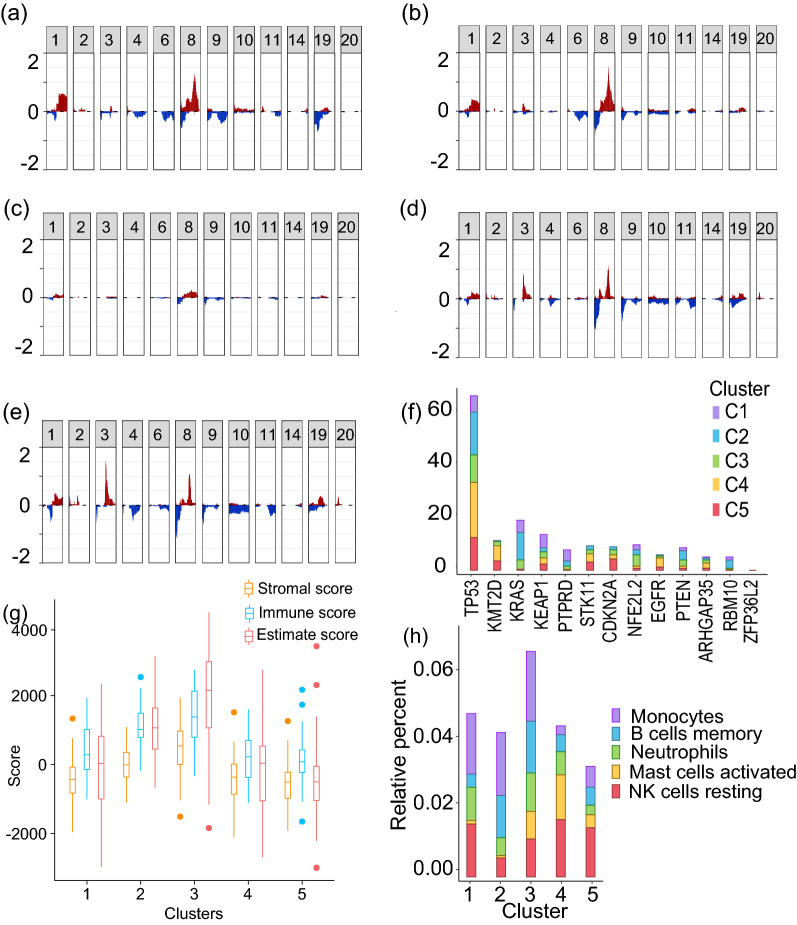
Molecular characters of samples with class labels obtained using methylation data. (**a**)–(**e**) Frequency plots for copy number variation corresponding to clusters 1–5 (y-axis: proportion of copy number gain/loss, x-axis: Chromosome number) and (**f**) Mutation of driver genes in the subgroups. (**g**) Box plot showing the distribution of stromal, immune, and ESTIMATE scores in each subgroup. (**h**) Bar plot showing the distribution of significantly enriched immune cell types in the subgroups.

To further validate the classification models, we used those samples for which only the methylation data was available. These samples were not used for cluster identification or classification as other levels of evidence were not available (i.e., incomplete data samples with respect to other levels of evidence). We obtained the subgroup label for these samples using the single-omic methylation non-linear decision-level fused model, as this model had the highest classification accuracy for single-omic data. The overall molecular characteristics of these samples, as expected, followed a similar trend as other samples. The samples in cluster 3 had the least copy number and mutational changes, and the highest immune cell infiltration (Fig. 6). This highlights that the proposed model can be used for the identification of the subgroups even in the case of incomplete data.

## Discussion

Subgroup identification is required for better management and treatment of cancer patients^3–5^. The availability of various molecular features as a consequence of the advancements in high-throughput genomic technologies has enabled the better subgrouping of cancer patients. We know that the phenotype of a patient is the resultant of various molecular features interacting non-linearly. To exploit this non-linear relation of molecular features, we used machine learning (ML) based methods. We used mRNA (F1), miRNA (F2), methylation (F3), and protein expression (F4) data from NSCLC samples. The latent representation of this multi-omics data was obtained using AE, a non-linear dimensionality reduction technique. This hidden representation was then clustered using consensus *K*-means clustering to identify five clusters. The clusters obtained with autoencoder (AE) based clustering were better than those obtained by clustering the preprocessed molecular features directly (Table 1). This indicates that AE was able to capture the interaction between the different levels of evidence effectively. We also showed that the AE-based clusters were more stable than the ones obtained using PCA, suggesting non-linear interaction between the molecular features (Table 1). Further, biological and clinical characterization of the clusters showed that cluster 3 showed better survival than other subgroups (Fig. 2f and g). This could be due to fewer genetic and epigenetic aberrations in the subgroup (Fig. 4). Two subgroups, cluster 1 and cluster 2, which had more LUAD patients showed poor survival, high genetic aberration, and also lower immune infiltration suggesting the highly aggressive nature of these tumors (Fig. 3 and Fig. 4).

ML based classification models (SVM, RF, and FFNN) were built using each level of evidence to predict the class labels. Linear and non-linear decision-level fused models were used to integrate the prediction probabilities from different classifiers and obtain the final subgroup label. DNA methylation (F3) based model had the best predictive ability among all (Table 2). DNA methylation carries epigenetic information, which is shown to play a vital role in cancer progression, metastasis, and prognosis. As different levels of evidence convey complementary information and work in conjunction, molecular features from different omic levels were fused at the feature-level to train the ML models. The combination of epigenetic information with proteomic information gave the best results in our experimental setup (Table 2). This suggests that protein expression carries more information than other single-omic levels. To the best of our knowledge, this is the first study proving that the combination of methylation and protein expression outperforms the other combinations. The model trained with feature-level fusion performed better than that with individual levels of evidence, and the decision-level fused model performed better than individual classification models. These results confirmed our hypothesis that the phenotype is the resultant of a combination of molecular features across different omics. The better performance of the linear decision-level fused model when compared to the non-linear decision-level fused model may be attributed to the less number of samples available to train the $L_1$ non-linear classifiers. The decision-level fused models trained using the features from the autoencoder (F$_{AE}$) have high classification accuracy (Table 2 and Fig. 5). One of the reasons for the better performance of the AE-based features, besides the ability of AE to capture the variation in the data, might be attributed to the fact that the classification labels were obtained by clustering the F$_{AE}$. Also, the ML algorithms were able to effectively model the class-specific decision boundaries generated by the clustering algorithm.

To summarise, this work proposed an end-to-end pipeline for machine learning-based subgroup identification in non-small cell lung cancer (NSCLC). We also proposed and validated the fusion-based classification models for the identification of subgroups in new samples. Since the classification models were built for individual levels of evidence, they can be used in the presence of single omic data as well. The generalizability of our model is yet to be validated due to the limitation in terms of the availability of an independent dataset. Also, exposure to more samples both in terms of heterogeneity and the number of samples, might provide better insights into the resulting subgroups. Therefore, the future work would include validating the proposed method in an independent cohort of data.

The performance in the current work is based on several assumptions made at different levels. These include preprocessing of the data to reduce dimensionality, using the most well-known ML models, and using cluster labels for subgroup identification. All these need independent evaluation, which may further help to better understand the non-linear processing happening in ML. Also, the better unearthing of biological knowledge using ML models. The comparable performance of regular *K*-means and GMM with consensus *K*-means in terms of Silhouette coefficient and Calinski Harabasz index needs further analysis and will be considered for future studies. Further, including additional information from whole slide histopathological (H and E) images as an additional level of evidence can provide better insights.

## Materials and methods

### Datasets and data preprocessing

The proposed pipeline was applied on the TCGA NSCLC (LUAD and LUSC) samples. TCGA multi-omics data comprising mRNA, miRNA, methylation, mutation, and copy number variation were downloaded from the GDC data portal. *TCGAbiolinks* (v 2.18.0) package in R^46^ was used to obtain this data for samples from LUAD and LUSC tumor types. Protein expression (RPPA level - 4) data was downloaded from the TCPA data portal^47,48^. Further, cBioPortal^49^ was used to obtain the clinical data. In this study, each level of evidence (single-omic) is referred to as a factor. The mapping from omic levels to the factors is shown in Supplementary Table S1. In the initial part of this work, only the samples which had data from all the four levels of evidence were considered.

It can be observed from Supplementary Table S1 that the dimension of data (*p*) was high compared to the number of samples (*n*). Hence, the preprocessing of data was carried out to ensure reliability besides reducing the dimension of the data^27,50^. Preprocessing of raw data which included, selecting a subset of features, imputing the missing values, and data transformation, was carried out as outlined in Supplementary Figure S1. All the protocols followed to carry out the preprocessing were obtained from previous studies^16,20,33,50,51^.

Briefly, in the case of F1 (FPKM values of protein coding mRNAs) and F2 (RPKM values of miRNAs), genes with zero expression in more than $20\%$ of the samples were dropped^16^. Genes in F1 were then sorted based on the standard deviation, and the top 2000 most variable genes were considered for further analysis^33^. Features retained in both the cases were scaled by min-max normalization to ensure that the data ranged between the values of 0 and 1. In the case of F3 (DNA methylation), beta values were used for analysis. The CpG probes on X and Y chromosomes, those mapping to SNPs or cross hybridized were dropped. The preprocessing was carried out using the *DMRCrate* (v 2.4.0) package^52^ in R. Samples and probes with more than $10\%$ of the data missing were dropped^20,33,50^. Further, the NAs in the retained probes were imputed using K-nearest neighbors (KNN) (K = 5)^20,33,50^. The selected probes were then sorted in the decreasing order based on their standard deviation and the top 2000 probes were considered for further analysis^33^. As beta values range from 0 to 1, further normalization was not required. For F4 (protein expression level-4), proteins whose expression was missing in more than $10\%$ of the samples were dropped. And as before, the missing values in the retained dimensions were imputed by KNN (K = 5). Normalization was not needed in the case of F4, as level-4 data was already normalized.

The preprocessed features corresponding to the feature-vectors (samples) common across all the four different levels of evidence (F1–F4) were stacked to obtain the multi-omics data matrix (Fig. 1 a, Supplementary Table S1, and Supplementary Tables S11–S15). This multi-omics matrix was then used further for dimensionality reduction (Fig. 1 a).

### Multi-omics data integration and cluster identification

Even after selecting the subset of features by preprocessing, the dimensionality (*p*) of the various factors was still high compared to the sample size (*n*). This ($\,p>> \,n$) may lead to overfitting when modeled using machine learning algorithms^27^. We also know that the biological features from different levels of evidence interact non-linearly to produce the final cancer phenotype^17,18^. Hence, to reduce the dimension of multi-omics data by retaining the non-linear interaction among the biological features, we used an autoencoder (AE) (Fig. 1b)^16,20^.

Multi-omics data was split with the train-validation split of 90–10% and used to train the AE model. The AE model was trained for 100 epochs with early stopping criteria, i.e., the model training was stopped if the validation error did not reduce for five subsequent epochs. The input data was fed in batches of 24 samples each. Rectified linear unit (ReLU) was used as the activation function, mean-squared error (MSE) as the loss function, and adaptive moment estimation (Adam) as an optimizer, as the input data was continuous. The AE model was built using the *KERAS* (2.4.0) library in Python 3 in Google Colab.

Different architectures of AEs were obtained by varying the number of layers, and the number of nodes in each layer. The performance of AE model was measured in terms of training and validation loss (Supplementary Table S2). The model tends to overfit the data when the difference between the training and validation loss is large^19^. Hence, the model which had the smallest difference between the training and validation loss was considered for subsequent analysis.

The lower-dimensional representation of the multi-omics data was obtained from the bottleneck layer of the trained AE model (Fig. 1b). Consensus *K*-means clustering was then applied to this representation to identify the clusters (Fig. 1c)^33,53^. Cluster labels were obtained for different number of clusters (*K*) by varying *K* from 2 to 10. The process of clustering was repeated 1000 times using $80\%$ of the samples each time^33^. The most consistent cluster was identified based on the proportion of ambiguously clustered pairs (PAC). This metric is quantified with the aid of the cumulative distribution function (CDF) curve^54^. The section lying in between the two extremes of the CDF curve ($u_1$ and $u_2$, Supplementary Figure 2a) quantifies the proportion of samples that were assigned to different clusters in each iteration. PAC is used to estimate the value of this section. It represents the ambiguous assignments and is defined by Eq. (1), where *K* is the desired number of clusters.

1 $$\begin{aligned} PAC_K = CDF_K(u_2) - CDF_K(u_1). \end{aligned}$$

Lower the value of PAC, lower the disagreement in clustering during different iterations, or in other words, more stable are the clusters obtained^54^.

### Characterization of clusters

To determine if there exists any difference in the survival between the clusters obtained, Kaplan-Meier (KM) survival curves and log-rank test were used (Fig. 1d). The end points for survival analysis was defined by overall survival (OS) and disease-free survival (DFS). OS is defined as the period from the day of initial diagnosis till death. DFS is defined as the time period from the day of treatment till the first recurrence of tumor in the same organ^55^. Survival analysis was carried out in R using the *Survival* (v 3.2-7) package.

To identify the features specific to each cluster in each level of evidence, feature selection was carried out by statistical tests as described in Supplementary Figure S5^20,33^. To summarize, the features with zero expression in more than $20\%$ of the samples in F1, F2, and F4, were dropped. To identify the differentially expressed (DE) features describing each subgroup, ANOVA with Tukey’s post-hoc test was used. In the case of F3, preprocessing was carried out as mentioned before (section: Datasets and data preprocessing). Further, the probes with standard deviation of more than 0.2 were quantile normalized, $log_2$ transformed, and limma was used to compare the expression of probes (Supplementary Figure S5). Additionally, mutation and copy number variation data were also used to characterize each cluster. A binary mutation matrix indicating the presence or absence of mutation in the driver genes was obtained. Fisher’s test was carried out on the driver genes with non-silent mutations. The genes with FDR $q~\le ~0.05$ were used for further interpretation. Copy number variation (CNV) data (segment mean) obtained from TCGA was analyzed using GISTIC 2.0^56^. The cytobands with $abs(SegMean)~\ge ~0.3$ were considered as altered and were subjected to Fisher’s test. The cytobands with $p~\le ~0.01$ were considered for characterization.

Immune, stromal, and estimate score for each sample was obtained from ESTIMATE analysis^42^ and subjected to ANOVA. CIBERSORT analysis was carried out using the LM22 signature gene set^43^. ANOVA with Tukey’s post-hoc test was carried out on these immune cells, and those with $log_2(FoldChange)\ge 1$ and $q\le 0.05$ were considered for further interpretation of the characteristics of each cluster. Gene Set Enrichment Analysis (GSEA) was also carried out using the Hallmark signature gene sets obtained from MSigDB^44,45^. The expression data from all the protein-coding genes were used as input for GSEA analysis.

### Subgroup identification by classifier combination

Classification models were built to identify the subgroup to which a new sample will belong. Three supervised classification models ($L_0$), support vector machine (SVM), Random forest (RF), and feed-forward neural network (FFNN) were built separately for each single-omic level. These models were trained using the class labels obtained from consensus *K*-means clustering as output labels. The input to the models were the molecular features specific to each subgroup (DE features) selected from individual omic levels (as described in previous section and Supplementary Figure S5 and Supplementary Tables S16–S19). The train-test split of 90–10% was used to build these models.

As the data was non-linearly separable, a radial kernel was used for SVM. The hyperparameters for SVM and RF were obtained by 5-fold cross-validation (CV) repeated ten times. For the FFNN, appropriate number of layers and neurons were chosen based on the dimension of the input vector. Categorical cross-entropy was used as the loss function with Adam optimizer while training the FFNN. To avoid overfitting, each fully connected layer was followed by a dropout layer (0.1), and *L*2 activity regularizer (1e-04) and *L*1 weight regularizer (1e-05). The models were trained with different learning rates (0.1, 1e-02, 1e-03, 1e-04, and 1e-05), and the one with the best accuracy was chosen.

To obtain an unambiguous prediction model, the prediction probabilities from each of these classifiers ($P_{SVM}$, $P_{RF}$, and $P_{FFNN}$) were concatenated and a new representation ($P_{C}$) was obtained. Decision-level fused classifiers ($L_1$) were built with this new feature representation as input and subgroup labels obtained by clustering as the target. The prediction probabilities were combined linearly and non-linearly to obtain linear and non-linear decision-level fused classifiers (Supplementary Figure S6).

In the case of linear decision-level fused model, the prediction probabilities obtained from $L_0$ models ($P_{SVM}$, $P_{RF}$, and $P_{FFNN}$) were weighted by $\alpha$, $\beta$, and $\gamma$, respectively^17,29^. The final classification probability ($P_{L}$) was obtained by the weighted summation of individual prediction probabilities using Eq. (2)^57^.

2 $$\begin{aligned} P_{L} = \alpha \times P_{SVM} + \beta \times P_{RF} + \gamma \times P_{FFNN}. \end{aligned}$$

The values of $\alpha$, $\beta$, and $\gamma$ were varied from 0 to 1 in steps of 0.05 by ensuring that they sum up to 1 (Supplementary Algorithm I).

In the case of the non-linear decision level fused model, the concatenated prediction probabilities ($P_{C}$) from the $L_0$ models were used to train the non-linear classifiers like logistic regression (LR) and FFNN to identify the subgroup labels^58^. Here, two non-linear decision-level fused models with different train-test splits were trained. In the first model, both $L_0$ and $L_1$ learners were trained with the complete training data set (without holdout). For the second model, a hold-out set was created by splitting the training data set. Here, the $L_0$ learners were trained using $60\%$, and $L_1$ learners using $40\%$ of the training data set.

As different levels of evidence carry complementary information, the combination of features from different omic levels will provide additional insights. Hence, the technique of feature-level fusion can help in better classification^17,29^. Here, features from different molecular levels were concatenated to obtain a new feature representation. This fused representation was then used to train each of the ML classifiers.

## Supplementary Information

Supplementary Information 2.

Supplementary Information 2.

Supplementary Information 3.

## Data Availability

All datasets used in this study are publicly available. The preprocessed data used to identify the subgroups is attached as the supplementary material (Supplementary Tables S11, S12, S13, S14 and S15). The data used to train the classification models is also attached as the supplementary material (Supplementary Tables S16, S17, S18, and S19). Raw data be downloaded from the following websites: Genomic Data Commons Data Portal (https://portal.gdc.cancer.gov/repository?facetTab=cases&filters=%7B%22op%22%3A%22and%22%2C%22content%22%3A%5B%7B%22op%22%3A%22in%22%2C%22content%22%3A%7B%22field%22%3A%22cases.project.project_id%22%2C%22value%22%3A%5B%22TCGA-LUAD%22%2C%22TCGA-LUSC%22%5D%7D%7D%5D%7D), obtain the manifest file using the link and use the GDC Data Transfer Tool to download the files. (https://gdc.cancer.gov/access-data/gdc-data-transfer-tool). The Cancer Proteome Atlas ( https://tcpaportal.org/tcpa/download.html), chose LUAD and LUSC (level-4) as projects and click download. cBioPortal for Cancer Genomics (https://www.cbioportal.org/study/clinicalData?id=luad_tcga_pan_can_atlas_2018%2Clusc_tcga_pan_can_atlas_2018), click on download button to download the data.

## Code availability

All the codes used in this work are available on GitHub: https://github.com/seemark11/NSCLC-subgroup-identification.
