# Supplementary material for: Machine learning based combination of multi-omics data for subgroup identification in non-small cell lung cancer
Source: Sci Rep. 2023 Mar 21;13:4636. doi: 10.1038/s41598-023-31426-w (PMC10030850; doi:10.1038/s41598-023-31426-w)
Supplement: Supplementary file 1 — Supplementary Information 2. [file 41598_2023_31426_MOESM1_ESM.pdf]

Supplementary Material

Machine Learning Based Combination of Multi-omics Data for Subgroup Identification in Non-small Cell Lung Cancer

Seema Khadirnaikar[1], Sudhanshu Shukla[2,*], and S R M Prasanna[1]
[1]Department of Electrical Engineering, Indian Institute of Technology Dharwad, Dharwad, India
[2]Department of Biosciences and Bioengineering, Indian Institute of Technology Dharwad, Dharwad, India
***sudhanshu@iitdh.ac.in**

Supplementary Table S1: Summarizing the levels of evidence, their mapping, number of samples, original dimension, and dimensions retained after preprocessing.

Omic type	Factor	Number of samples	Original dimension	Dimension retained
mRNA	Factor 1 (F1)	1014	19,514	2000
miRNA	Factor 2 (F2)	991	1881	407
DNA methylation	Factor 3 (F3)	828	485k	2000
Protein expression	Factor 4 (F4)	687	237	216
		566 *	544k	4623

Note: k here indicates the multiple of 1000.
* - samples common across all the levels of evidence

Supplementary Table S2: Summarizing the training and validation losses for different autoencoder (AE) architectures.

Sl. No.	Nodes in hidden layer	Nodes in bottleneck layer	Train Loss	Val Loss	Difference
1	2000, 1000	500	0.026	0.034	0.008
2	2000, 1000	400	0.029	0.035	0.006
3	2000, 1000	300	0.024	0.034	0.01
4	2000, 1000	200	0.025	0.035	0.01
5	1000	100	0.022	0.032	0.01
6	1000, 500	100	0.027	0.034	0.007
7	2000, 1000, 500	100	0.033	0.037	0.004
8	2000, 1000, 500	50	0.03	0.036	0.006
9	2000, 1000, 500, 100	50	0.032	0.038	0.006
10	2000, 1000, 500	25	0.031	0.037	0.006

Supplementary Table S3: Comparing the clustering scores between the clusters (*K* = 5) obtained using iClusterPlus and the proposed technique applied on the multi-omics data.

Omic	Technique	Silhouette coefficient	Calinski Harabasz index
**Multi-omics**	iClusterPlus	0.044	37.791
**Multi-omics**	Proposed technique	0.214	163.322

Supplementary Table S4: Comparing the Silhouette coefficient and Calinski Harabasz index for clusters obtained using different clustering algorithms (*K* = 5).

Clustering Algorithm	Silhouette coefficient	Calinski Harabasz index
**Consensus k-means**	0.214	163.322
**HC**	0.093	20.242
**GMM**	0.202	131.376
**k-means**	0.213	163.404

Supplementary Table S5: PAC values obtained (K = 5) after clustering the reduced dimension data obtained from AE trained with multi-omic data obtained by varying the input dimensions from F1 and F3 levels (F2 = 407 and F4 = 216).

Dimension of F1 and F3	Multi-omic data dimension	Architecture of AE	PAC (K = 5)
1000	2623	2000, 1000, 500, 100	0.32
		1000, 500, 100	0.35
3000	6623	4000, 2000, 1000, 500, 100	0.28
		3000, 1000, 500, 100	0.35
4000	8623	4000, 2000, 1000, 500, 100	0.27
		3000, 1000, 500, 100	0.35
2000	4623	2000, 1000, 500, 100	0.14

Supplementary Table S6: Summarizing the number of dimensions retained after applying filters for statistical tests, and number of dimensions retained after statistical analysis at each molecular level.

	**Original Dimension**	**Filtering Condition**	**Dimensions retained after filtering**	**Dimensions retained after statistical test**
**Factor 1 (Lnc RNA)**	12719	Zero <= 20% samples	4364	126
**Factor 1 (PcGs)**	19514	Zero <= 20% samples	16524	672
**Factor 2 (miRNA)**	1881	Zero <= 20% samples	432	9
**Factor 3 (methylation)**	485k	Standard deviation >= 0.2	8488	719
**Factor 4 (protein expression)**	237	Zero <= 20% samples	216	153
**Factor 5 (Driver mutation)**	298	Mutated in 1% samples	220	13
**Factor 6 (Amplified cytoband)**	33	pval <= 0.01	20	14
**Factor 6 (Deleted cytoband)**	52	pval <= 0.01	35	11

Supplementary Table S7: Significantly enriched pathways obtained from Metascape in each subgroup.

**C1**	**C2**	**C3**	**C4**	**C5**
Hormone secretion and transportation	Negative regulation of hydrolase activity	Cellular extravasation and cilium movement	Epidermis development	Paraxial mesoderm development
Regulation of hormone levels	Organic acid, monocarboxylic acid and carboxylic acid biosynthetic process	Chemokine production and chemotaxis	Keratinocyte differentiation	Establishment or maintenance of epithelial cell apical/basal polarity, apical/basal cell polarity, and bipolar cell polarity
Peptide, protein and insulin secretion	Negative regulation of phosphoprotein phosphatase activity and protein dephosphorylation	Leukocyte mediated cytotoxicity	Peptide cross-linking	Presynapse assembly and organization
Carboxylic acid, organic acid, organic anion, and monocarboxylic acid transport		Regulation of leukocyte cell-cell adhesion	Synaptic signaling	Negative regulation of BMP signaling pathway and cellular response to growth factor stimulus
Regulation of ion transport, wound healing		Negative regulation of cytokine production	Regulation of peptidase activity	Regulation of neuron death

Supplementary Table S8: Summarizing the features specific to each subgroup.

	C1	C2	C3	C4	C5
**Survival**		Worst OS and DFS	Best OS		BEST DFS
**Driver mutation**	lower mutation rate of CDKN2A, NFE2L2, PTEN, ZFP36L2	lower mutation rate of CDKN2A, NFE2L2, PTEN, ZFP36L2, ARGHGAP35	lower mutation rate of RBM10, NFE2L2, ZFP36L2, ARHGAP35	lower mutation rate of EGFR, KRAS, STK11, RBM10	lower mutation rate of EGFR, KRAS, STK11, RBM10, ZFP36L2
**Copy number alteration**	Amplification of Chr8	Amplification of Chr8	Less alteration overall		
**Tumor micro environment**			Higher stromal score, immune score and estimate score		
**Immune cells**		Higher infiltration of Monocytes	Higher infiltration of B cells memory and Monocytes	Higher infiltration of Mast cells activated and Neutrophils	Higher infiltration of NK cells resting and Mast cells activated

Supplementary Table S9: Gene sets significantly (FDR q ≤ 0.05) positively enriched in hallmark gene set in C3 vs. rest analysis

NAME	ES	NES	NOM p-val	FDR q-val	FWER p-val
HALLMARK_INFLAMMATORY_RESPONSE	0.616	2.151	0	0.005	0.006
HALLMARK_COMPLEMENT	0.560	2.147	0	0.003	0.006
HALLMARK_IL6_JAK_STAT3_SIGNALING	0.630	2.093	0	0.003	0.010
HALLMARK_ALLOGRAFT_REJECTION	0.671	2.086	0	0.003	0.011
HALLMARK_KRAS_SIGNALING_UP	0.484	1.985	0	0.006	0.027
HALLMARK_COAGULATION	0.528	1.977	0.002	0.006	0.031
HALLMARK_INTERFERON_GAMMA_RESPONSE	0.651	1.937	0.012	0.008	0.048
HALLMARK_IL2_STAT5_SIGNALING	0.430	1.819	0.002	0.019	0.118
HALLMARK_TNFA_SIGNALING_VIA_NFKB	0.490	1.742	0.034	0.033	0.191
HALLMARK_INTERFERON_ALPHA_RESPONSE	0.624	1.653	0.071	0.058	0.329

Supplementary Table S10: Gene sets significantly (FDR q ≤ 0.05) negatively enriched in hallmark gene set in C3 vs. rest analysis

NAME	ES	NES	NOM p-val	FDR q-val	FWER p-val
HALLMARK_G2M_CHECKPOINT	-0.751	-2.147	0	0.0008	0.001
HALLMARK_MYC_TARGETS_V1	-0.724	-2.118	0	0.001	0.003
HALLMARK_E2F_TARGETS	-0.778	-2.111	0	0.001	0.005
HALLMARK_MYC_TARGETS_V2	-0.789	-2.091	0	0.001	0.006
HALLMARK_DNA_REPAIR	-0.496	-1.912	0.004	0.014	0.050
HALLMARK_MTORC1_SIGNALING	-0.521	-1.838	0.004	0.025	0.107
HALLMARK_UNFOLDED_PROTEIN_RESPONSE	-0.472	-1.818	0.004	0.027	0.131

Supplementary Figures

Supplementary Figure S1: Flowchart summarizing the steps involved in preprocessing and selecting a subset of features for dimensionality reduction.

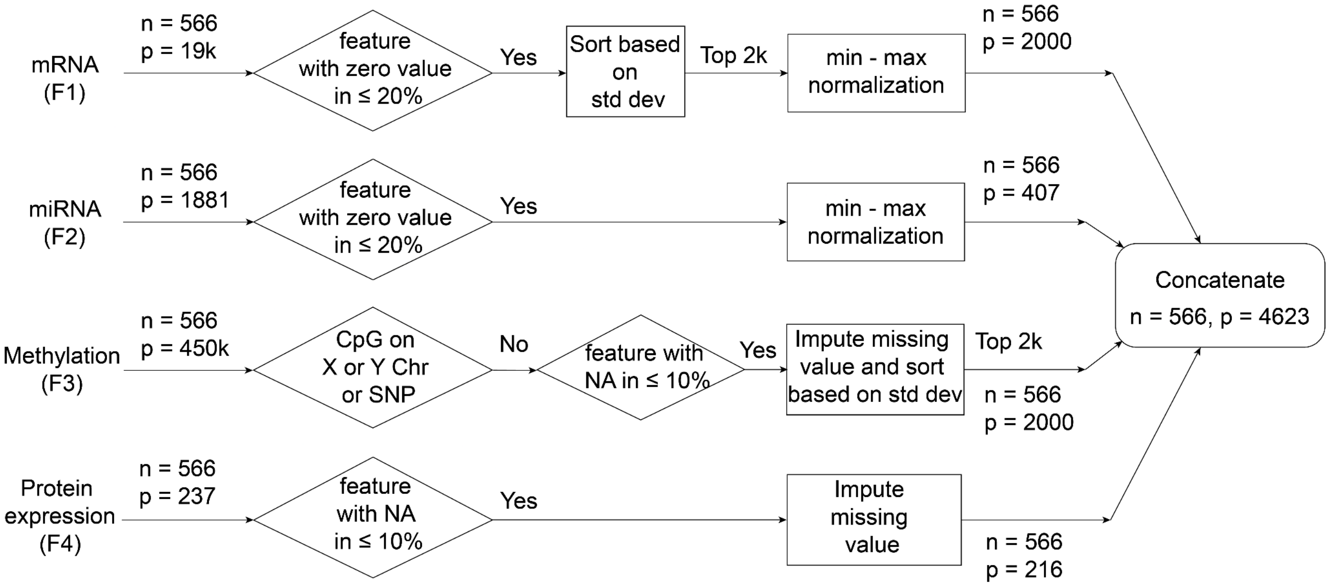

Supplementary Figure S2: Training and validation losses of autoencoder.

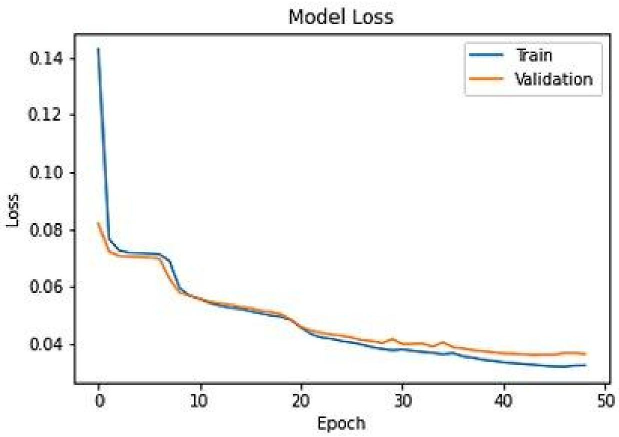

Supplementary Figure S3: Consensus clusters obtained for k = 2 **(a)** CDF curves for consensus clustering (K = 2 to K = 10) **(b)** Consensus heatmap for k = 2, and **(c)** Bar plot showing the distribution of histological subtypes of NSCLC (LUAD and LUSC) in clusters obtained from consensus k-means clustering (k = 2).

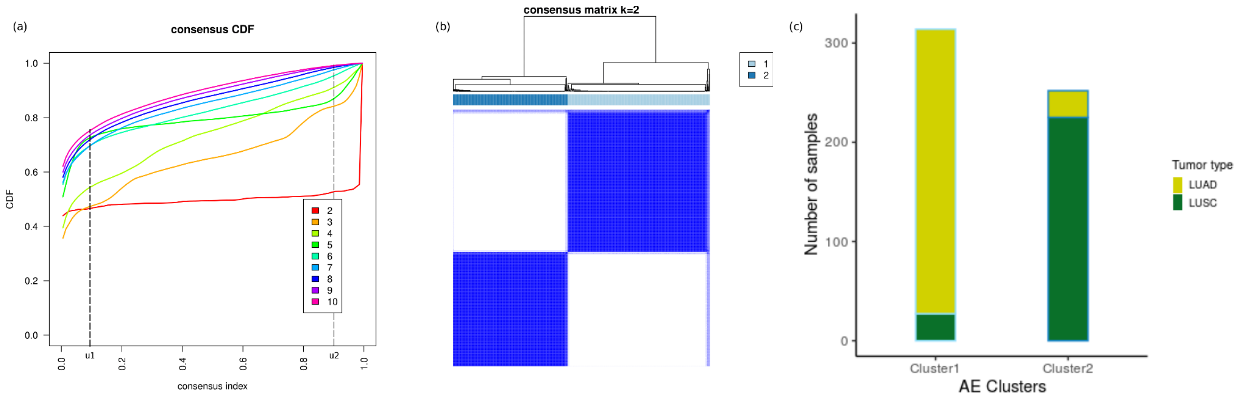

Supplementary Figure S4: UMAP plots showing the distribution of samples **(a)** before and **(b)** after dimensionality reduction using autoencoder (AE). Samples are colored based on the labels obtained from consensus k-means clustering.

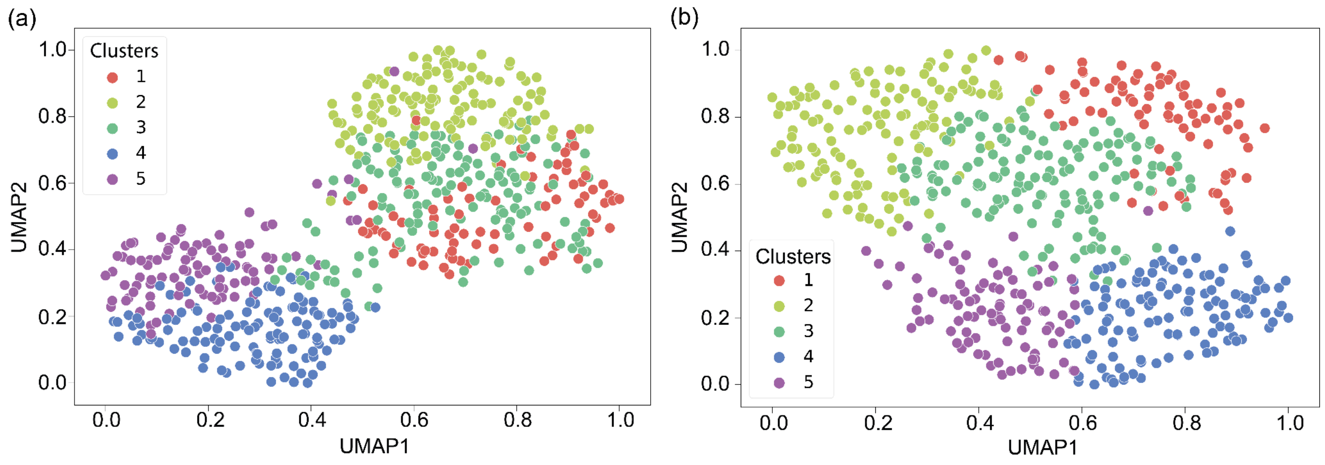

Supplementary Figure S5: Flowchart summarizing the steps involved in preprocessing the features for statistical tests, and features retained after statistical tests to draw biological inferences and to train the ML models.

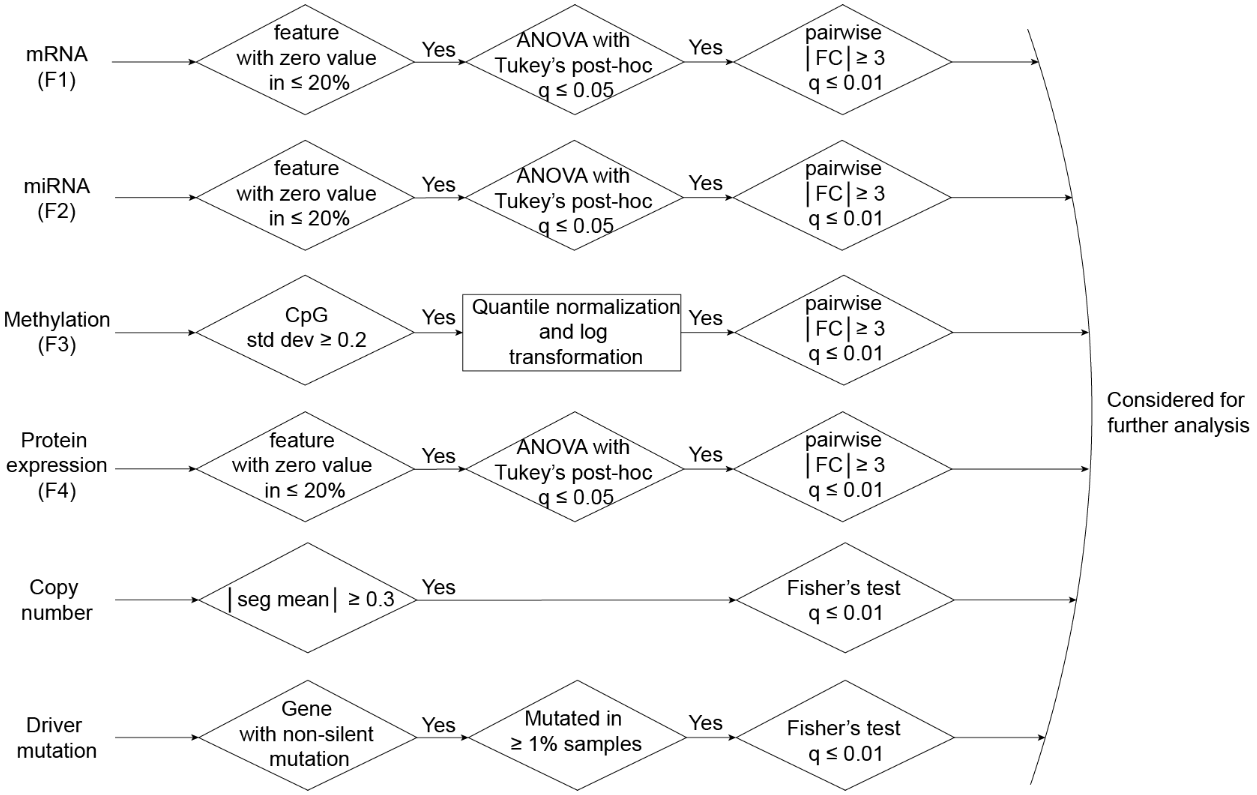

Supplementary Figure S6: Overview of ensemble models built with linear and non-linear combination of base learners (SVM: support vector machine, RF: random forest, FFNN: feed forward neural network, log reg: logistic regression).

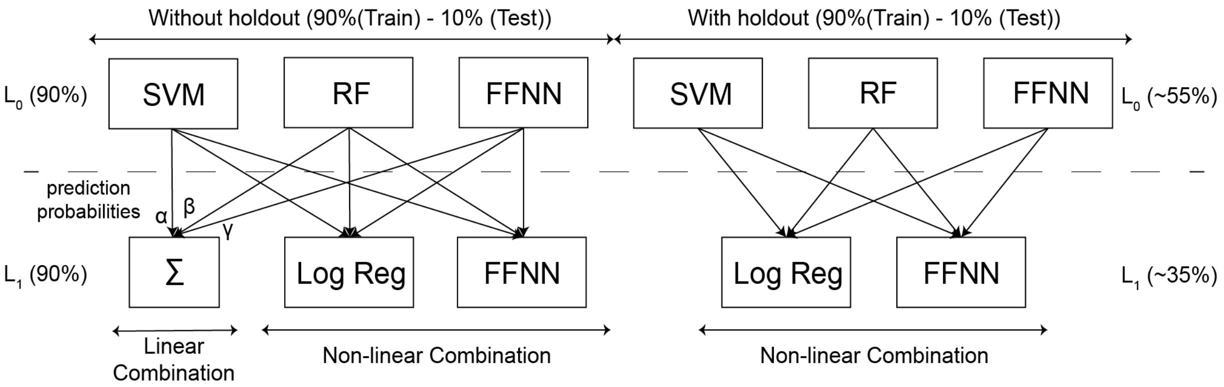

The algorithm used to compute the values of α, β, and γ **needed to compute the prediction probabilities for decision-level fusion of linear ensemble model.**

Supplementary Algorithm 1: Calculate α, β, and γ

$\alpha = 0{:}\ 0.05{:}\ 1$

for α_i in α:

  $\beta = 0 : 0.05 : (1 - \alpha_i)$

  for β_j in β:

    $\gamma = 1 - \alpha_i - \beta_j$

  end for

end for
